# SNPConvert: SNP Array Standardization and Integration in Livestock Species

**DOI:** 10.3390/microarrays5020017

**Published:** 2016-06-09

**Authors:** Ezequiel Luis Nicolazzi, Gabriele Marras, Alessandra Stella

**Affiliations:** 1 Bioinformatics Core Facility, PTP Science Park, Via Einstein—Loc. Cascina Codazza 26900 Lodi, Italy; gabriele.marras@ptp.it (G.M.); stella@ibba.cnr.it (A.S.); 2 Istituto di Biologia e Biotecnologia Agraria—Consiglio Nazionale della Ricerca, Via Einstein—Loc. Cascina Codazza 26900 Lodi, Italy

**Keywords:** single nucleotide polymorphism, array, software, standardization, integration

## Abstract

One of the main advantages of single nucleotide polymorphism (SNP) array technology is providing genotype calls for a specific number of SNP markers at a relatively low cost. Since its first application in animal genetics, the number of available SNP arrays for each species has been constantly increasing. However, conversely to that observed in whole genome sequence data analysis, SNP array data does not have a common set of file formats or coding conventions for allele calling. Therefore, the standardization and integration of SNP array data from multiple sources have become an obstacle, especially for users with basic or no programming skills. Here, we describe the difficulties related to handling SNP array data, focusing on file formats, SNP allele coding, and mapping. We also present SNPConvert suite, a multi-platform, open-source, and user-friendly set of tools to overcome these issues. This tool, which can be integrated with open-source and open-access tools already available, is a first step towards an integrated system to standardize and integrate any type of raw SNP array data. The tool is available at: https://github. com/nicolazzie/SNPConvert.git.

## 1. Introduction

For over fifty years, “traditional” livestock breeding has combined phenotypes and statistical models to infer the genetic value of individuals. Since its first application, SNP array technology revolutionized this scenario for the animal genetics industry and research. In fact, for the first time, it was possible to obtain consistent and cost-effective genetic information directly from dense genome-wide markers. A review of the impact of the genomic revolution on domestic animals is presented in [1]. The interest raised towards such technology created new opportunities for companies providing these service (e.g., Illumina and Affymetrix). In a short time, a large series of products were produced to fit market requests. As a consequence, a wide variety of low-, medium- and high-density SNP arrays, and their updates carrying slight modifications in the number and content of SNPs, were marketed. Unfortunately, this rapid expansion of availability of genomic information was not matched by a collective effort to standardize data, file formats, or type of information provided to end-users. In cattle livestock alone, three companies have produced more than 15 commercial SNP arrays in less than 10 years, and this number would more than double if the “private” SNP arrays (e.g., those produced by specific companies/consortia but not openly accessible to the public) were considered.

Each genotyping platform is associated to a specific genotyping software to produce a range of output formats. By default, Illumina provides two possible file formats: the row format (where all the information for each SNP for each individual is provided on each row) and the matrix format (where the genotype call of each SNP for all individuals is provided on each row). The “ExampleData” folder of this tool, provides examples for each type of data. Using specific plug-ins, other output formats can also be accommodated. Affymetrix’s genotyping software for Windows users returns a range of output formats, whereas only the Affymetrix format is available for Linux/Mac users (individuals by row and SNP dosage—0, 1, 2 corresponding to the number of “B” alleles—by column). Having full access to the software and raw data would make the standardization and integration of data relatively easy. However, a highly common scenario is that researchers and industry do not have access to raw data or the genotyping platform software, or simply exchange genomic data produced in multiple formats, which makes *post-hoc* standardization and integration of SNP data a difficult but essential need. Consistent SNP IDs, allele-coding formats (e.g., A/B, Top, Forward *etc.*), map positions in a specific reference genome assembly, and other required information were not readily accessible to the general public until very recently, when producers and researchers started collaborating and sharing information. This collaboration resulted in the “SNPchimp” tool [2], an open-access multi-species online database that stores all the above information from the source (e.g., producers) and ensures full user-friendly access to the information. SNPchimp is a strategic tool for data analysis, but custom programs are still needed to standardize genotype allele coding and map coordinates. Thus, although a step forward was made towards the standardization of SNP array data, effective standardization and integration of formats, allele coding and map information was still unaccounted for end users with limited or no programming skills.

Here, we present the SNPConvert suite, a simple set of programs to convert any Illumina raw file format (both row and matrix formats) to a PLINK basic format (see [3,4] for specifics on this format) and to modify the allele coding and update the genomic coordinates of any PLINK file, irrespectively of the technology used to produce the genotypes. This set of programs, released both as multi-platform source code intended for command line access and as a simplified graphical user interface (GUI) for Windows and Mac users, is able to solve most common problems any researcher, irrespectively of its programming skills, has to face when integrating multiple SNP array datasets. Although we report on file format conversion for the Illumina technology, similar tools are available for Affymetrix-based genotypes [5,6,7]. However, note that Affymetrix and Illumina data cannot be integrated directly, as SNP IDs and the allele calling process are not consistent in the two genotyping technologies.

## 2. Materials and Methods

**Figure 1:**
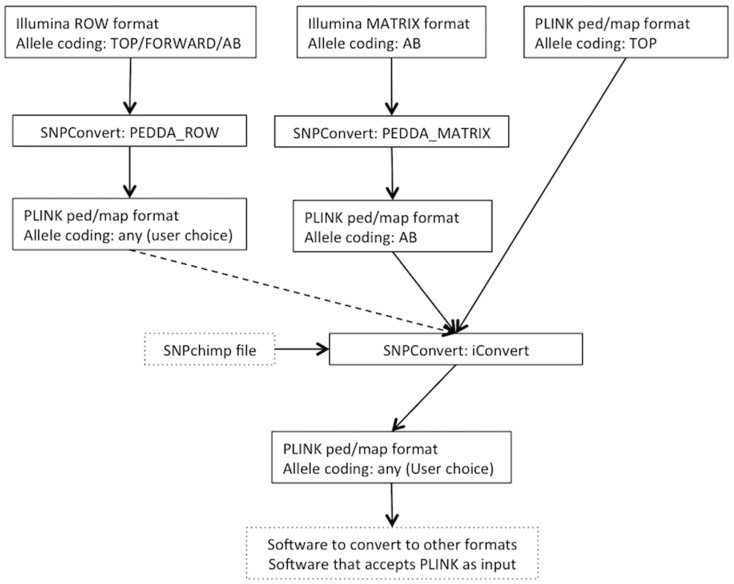
Flowchart of SNPConvert functionalities for files in Illumina row, matrix, or PLINK file format. Dashed lines indicate optional steps. Pointed boxes indicate interactions with third-party software.

The SNPConvert suite was designed and developed in Python 2.7. The suite contains a set of three Python utilities that convert any Illumina row and matrix raw file formats to PLINK format (“PEDDA_ROW” and “PEDDA_MATRIX”, respectively) and can automatically modify the allele coding format and update the genomic coordinates of any PLINK file (“iConvert”). All three programs are multi-platform (e.g., they run on any operative system). A deliberate design choice was to use only Python’s built-in libraries to avoid dependencies of the whole structure and thus enhance portability. The use is restricted, from the end user’s point of view, to the modification of a simplified parameter file and the execution of the program(s) from the command line. An example flowchart is shown in Figure 1.

### 2.1. Utility n.1: PEDDA_ROW

PEDDA_ROW software converts files in Illumina “row” format to PLINK (ped and map) format. To obtain this result, the user is asked to compile a parameter file to include the following:

(i) The paths to two input files: the FinalReport file (in row format) and the SNP map file, provided, together with a series of other files, by genotyping labs using the Illumina (H)iScan technology;

(ii) The selected allele coding output (generally, Illumina row file formats contain multiple allele coding);

(iii) The separator of the file (comma, tab or space);

(iv) The position of the SNP ID in the FinalReport file;

(v) The position of the Individual ID in the FinalReport file;

(vi) (Optional) the population ID (referred in PLINK as FID—family ID), displayed as the first column of the PED file;

(vii) (Optional) the output file name.

Based on these parameters, the software reads and processes/writes the genotypes (in the chosen allele coding format) of a single individual at a time (to reduce the amount of memory required).

### 2.2. Utility n.2: PEDDA_MATRIX

Similarly, the PEDDA_MATRIX software converts files into the Illumina “matrix” format to PLINK format. Since the matrix format is much more standardized and can only accommodate one type of allele coding, the number of parameters to be set by the end-user is reduced to 4: (i) the paths to the same two input files as in PEDDA_ROW, but in this case the FinalReport file should be in matrix format; (ii) the separator of the file (comma, tab or space); (iii) the optional population ID; and (iv) the optional output file name. In this case, considering all individual genotypes are present on each line, the software stores the genotypes of all individuals before writing the output, thus requiring a larger amount of memory.

### 2.3. Utility n.3: iConvert

The iConvert software converts allele coding formats (e.g., Illumina TOP/Illumina or Affymetrix FORWARD/Illumina or Affymetrix AB) and, if required, updates genomic coordinates from any PLINK file, irrespectively of the technology used. The only constraint is the use of a SNPchimp output file, which makes this tool directly accessible by the animal genetics community only. However, retrieving the required information and creating a file with a similar format to SNPchimp output (using a spreadsheet such as Microsoft Excel) can by-pass this drawback for species/chips not included in SNPchimp. Again, the user is asked to compile a parameter file to specify: (i) the PLINK PED and MAP input files; (ii) the missing values for genotypes in the PLINK file; (iii) the SNPchimp file containing the input and output allele formats and the genomic coordinates to be used (if required); (iv) the input and output allele formats (should be present in (iii)); (v) the choice of updating or not the genomic coordinates. This program simply produces updated PLINK PED and MAP files. Similarly to PEDDA_ROW, each individual is processed singularly; thus, the requirements in terms of memory are low.

In order to facilitate access to these tools for Windows and Mac users (64 bit processors only), updated versions of the three aforementioned tools were wrapped into a graphical user interface (GUI). PyQt v.4 [8], a Python binding to the QT cross-platform project [9], was used to design the GUI and its functionalities. PyInstaller [10] was then used to create the two executable released. All the source codes used to create the GUI are freely available. The GUI was named SNPConvert v1.0, and it is a simple graphical application of the three software previously described, with the only difference of a further reduced set of parameter. Each of the three programs provides a full runtime log, where the user can check that all the data is read and handled as expected. It is important to note that, for GUI users, Python 2.7 is not a dependency.

## 3. Results

SNPConvert is a publicly available set of user-friendly tools. Both source codes and GUI are provided, including source codes used to obtain the GUI.

Ease of installation and use is ensured by the fact that SNPConvert (GUI or source code) is multiplatform. It was coded using only built-in Python packages and has only one generic dependency (e.g., Python 2.7) when running programs from the command line. The open-source nature of the tools aims at enhancing community-driven development and enhancement of the capabilities of the software.

The only limitation of SNPConvert is the amount of random access and virtual memory available. Program requirements are highly variable depending on the file format (e.g., the program used) and the number of markers and individuals. For example, the amount of memory requirement of PEDDA_MATRIX or its GUI counterpart is higher than in PEDDA_ROW or iConvert. The reason is that because the file format contains multiple individual genotypes on a single row, PEDDA_MATRIX reads and stores in memory all genotypes of all individuals, writing the output at the end of the whole process. On the contrary, PEDDA_ROW has a much lower use of resources. In fact, since Illumina row format files store genotypes of each individual consecutively, PEDDA_ROW retains all the SNPs of a single individual in memory prior to writing the output file. Similarly, iConvert reads and writes converted alleles over single individuals; thus, the use of resources is limited.

Both source codes and GUI were tested on “average” datasets, obtaining satisfactory results for an average user. For example, a full plate (96 samples) in matrix form was converted in less than 5 s (wall time) on a MacOs with 8Gb RAM and a 2.8 GHz Intel Core i7 processor. Using the same computer, but converting 777,962 SNPS (BovineHD) genotyped over 40 individuals reported in a row format file (e.g., a 4.75 Gb file), wall time increased to 2 min and 56 s to produce the output. Finally, remapping SNPs, converting allele coding of 417 BovineHD genotyped individuals (e.g., ~424.4 million conversions) and producing new genotype files took 8 min and 14 s of wall time. No appreciable differences in wall time were observed when running the programs from source code or from the GUI.

## 4. Discussion

The set of tools reported here are able to solve most of the standardization issues of formats, allele coding, and mapping of Illumina SNP arrays. In addition, if combined with another already available open-source tools [3,4,5] or the proprietary Windows-only Affymetrix GenotypingConsole [11], such full standardization extends also to the Affymetrix technology. However, it is important to note that, currently, such user-friendly tools to standardize data without the need of programming skills or advanced bioinformatics tools are technology-specific. Since Illumina and Affymetrix are based on different technology, with different protocols to call and identify alleles, advanced bioinformatics methodologies must be applied when cross-technology standardization is required.

This tool specifically addresses users without (or with limited) programming skills, as advanced and skilled users usually code their own programs to overcome these difficulties. In fact, special effort was put into making this tool multi-platform and user-friendly. These tools were coded using only built-in libraries in order to minimize installation requirements. The trade-off of this choice is some loss in the efficiency of some of SNPConvert’ functionalities. For example, iConvert could perform much faster using a multi-processor approach, which would require the installation of specific Python libraries. A reasonable increase in wall time was not considered a high cost in spite of its ease of installation/use. In any case, since source codes are released (e.g., including source codes to create the GUI), advanced users can use these scripts as a starting point for more advanced (and performing) applications.

Storing data in PLINK format might not seem the best choice, especially considering that PLINK ped and map files are not the most efficient formats in terms of disk memory usage. Again, a trade-off between memory usage and easy (or easier) access to genomic analysis was considered. The choice of PLINK format as standard can be beneficial because users can perform a number of analyses based on the same format, irrespectively of the technology used to obtain the gentoypes. In fact, PLINK format is the only common format in output for Illumina and Affymetrix software. Furthermore, PLINK ped and map files allow direct access to any PLINK functionality and any tool accepting PLINK file formats as input file [12]. This means that any user, experienced or not, is able to readily perform data quality checks and even advanced analysis (with some limitations). Finally, multi-file format converters for genomic data, such as PGDspider [13], can convert PLINK file formats many any other file formats.

## 5. Conclusions

In this work, we describe a number of issues researchers have to face when dealing with standardization and integration of SNP array data. Researchers with programming skills can overcome these difficulties by programming their own methods. However, the issues described here can be a large obstacle for those researchers having low or no programming skills. Here, we present a simple, open-source, multi-platform, and user-friendly set of tools to overcome these difficulties. The tool is available at: https://github.com/nicolazzie/SNPConvert.git. Most SNPConvert functionalities address Illumina-specific issue; however, if combined with already available open-source software, the solutions presented here are able to solve the problems described for all genotyping technologies.

## Abbreviations

The following abbreviations are used in this manuscript:

SNP: Single nucleotide polymorphism
GUI: Graphical user interface
